# Effect of *CYP2C19* genetic polymorphism on the pharmacodynamics and clinical outcomes for patients treated with ticagrelor: a systematic review with qualitative and quantitative meta-analysis

**DOI:** 10.1186/s12872-022-02547-3

**Published:** 2022-03-17

**Authors:** Qiufen Xie, Qian Xiang, Zhiyan Liu, Guangyan Mu, Shuang Zhou, Zhuo Zhang, Lingyue Ma, Yanjun Gong, Jie Jiang, Yimin Cui

**Affiliations:** 1 Department of Pharmacy, Peking University First Hospital, No. 6, Dahongluochang Street, Xicheng District, Beijing, 100034 China; 2 Institue of Clinical Pharmacology, Peking University, No. 38, Xueyuan Road, Haidian District, Beijing, 100191 China; 3 Department of Cardiology, Peking University First Hospital, No. 8, Xi Shi Ku Street, Beijing, 100034 China

**Keywords:** Ticagrelor, Pharmacogenomics, *CYP2C19*, Pharmacodynamics, Clinical outcomes, Systematic review

## Abstract

**Background:**

Studies show inconsistent results regarding the impact of *CYP2C19* genotype on the pharmacodynamics (PD) and clinical outcomes of ticagrelor. With the implementation of genotype-guided individualized antiplatelet therapy, the association between *CYP2C19* polymorphism and the efficacy and safety of ticagrelor for patients is still worthy of exploring and studying.

**Methods:**

This systematic review protocol has been registered in the PROSPERO network (No. CRD 42020158920). Electronic databases of PubMed, EmBase, and the Cochrane Library were systematically searched from inception to January 6th, 2022 to select studies investigating the impact of *CYP2C19* genotype on PD and clinical outcomes of ticagrelor. The results were presented as odds ratio (OR) or weight mean difference with its 95% confidence interval (CI) by using the random-effects model. Trial sequential analysis (TSA) was used to control risk of random errors and detect the robustness of outcomes.

**Results:**

Eight studies recruited a total of 6405 patients treated with ticagrelor. Mostly trials reported no significant effect of any or no *CYP2C19* loss-of-function (LOF) allele (*2 or *3) on all the endpoints. Compared with no LOF allele carriers, subgroup analysis suggested any LOF allele in Asian patients was associated with a significant decreased risk of bleeding events (OR: 0.41; 95% CI: 0.22–0.75; *P* = 0.004). Furthermore, any LOF allele carriers didn’t yield any impact on the risk of MACEs (OR: 1.11; 95% CI: 0.76–1.64; *P* = 0.586), stroke (OR: 1.71; 95% CI: 0.99–2.96; *P* = 0.054), definite stent thrombosis (OR: 0.88; 95% CI: 0.17–4.60; *P* = 0.882), bleeding (OR: 0.63; 95% CI: 0.27–1.46; *P* = 0.281), myocardial infarction (OR: 0.81; 95% CI: 0.30–2.20; *P* = 0.682), and revascularization (OR: 0.81; 95% CI: 0.33–2.00; *P* = 0.649) in all patients. The results of TSA were indicated that more further trials would be required.

**Conclusions:**

This qualitative and quantitative study suggested Asian patients carrying any *CYP2C19* LOF allele might have a lower risk of bleeding events comparing with no LOF allele carriers when treated with ticagrelor. However, we did not prove an important role of *CYP2C19* genotype on the risk of PD and clinical endpoints in the whole cohort. In future, more large-scale prospective studies and more different ethnic populations should be included.

**Supplementary Information:**

The online version contains supplementary material available at 10.1186/s12872-022-02547-3.

## Background

Nowadays, cardio-cerebrovascular diseases are the leading cause of death, morbidity, and disability worldwide [1, 2]. Lower blood flow in coronary arteries, and disfunction or death for part of heart muscle are main causes resulted in acute coronary syndrome (ACS) [3–5]. Dual antiplatelet therapy (DAPT) with aspirin and P2Y12 inhibitors are recommended to prevent thromboembolic complications in ACS patients scheduled percutaneous coronary intervention (PCI) [6]. However, response to traditional antiplatelet drugs is inter-individual variable and associated with differ on-treatment platelet reactivity and clinical outcomes [6, 7].

As a newer potent P2Y12 inhibitor, ticagrelor could reversibly bind to the P2Y12 receptor, and quickly reach the peak time of plasma concentration (within 2.5 h). Compared with clopidogrel, ticagrelor could provide more potent platelet inhibition because of faster onset [7], and yield greater benefits on ischemic events for ACS patients. Besides, subgroup analysis of Asian population in SOCRATES trial indicated ticagrelor was better efficacy in reducing the risk of vascular events than aspirin in acute stroke or transient ischemic attack (TIA) [8]. To our knowledge, the effect of genetic polymorphisms on the pharmacokinetics (PK), pharmacodynamics (PD) and clinical outcomes of antiplatelet drugs are still being explored and studied [9]. Many studies have confirmed that *CYP2C19* polymorphisms carry an important predictor for clinical events in antiplatelet therapy on ACS patients after PCI [10]. Indeed, as is well known, ticagrelor is not being activated by CYP2C19 enzyme [7]. However, when we reviewed published or registered pharmacogenomics studies, we found that the association between *CYP2C19* polymorphism and ticagrelor was not always negative regarding to the previous knowledge. Some studies have shown that compared with no LOF carriers, *CYP2C19* LOF allele carriers reduced the risk of bleeding in Asian patients [11–13], especially Yu's results [12]. While studies in Caucasians revealed an increased tendency for bleeding in the LOF allele carriers [14]. Starting in 2017, our team initiated a prospective multi-center cohort study named Impact of Biomarkers on Pharmacokinetics and Pharmacodynamics of Ticagrelor (NCT03161002), to determine the genetic polymorphism in both Chinese healthy subjects and patients treated with ticagrelor. According to the 1-year follow-up results of 208 ACS patients, we also preliminarily found that a trend of decrease in bleeding events of *CYP2C19* LOF allele carriers. Besides, from the recent results including 175 healthy volunteers in detected *CYP2C19* SNPs, rs17885098 might significantly influence platelet aggregation through candidate genes analysis. These results would be published later. To our knowledge, individualized antiplatelet therapy depends on many aspects. The benefit effects of ticagrelor were balanced due to more expensive price, high discontinuation rate, increased risk of bleeding and other adverse effects such as dyspnea [15]. Chinese physicians often choose ticagrelor for patients even being *CYP2C19* fast metabolizers, considering the high risk of major adverse cardiovascular events (MACE) [12]. Based on the above inconsistent results and interesting discovery, whether *CYP2C19* polymorphism directly affects PD and clinical outcomes of ticagrelor remains to be further verified and explored.

Our current systematic review was performed based on available evidences to evaluate the association with *CYP2C19* genotype and platelet reactivity or clinical endpoints in patients with ticagrelor. So that we could provide a basis for efficacy and safety of antiplatelet therapy for no matter *CYP2C19* LOF or non-LOF allele carriers. We hope we could provide a new and non-ignorable viewpoint for mechanism exploration and pharmacogenomic research of ticagrelor in future [16, 17].

## Methods

### Data sources, search strategy, and selection criteria

The standard flow was guided by the Preferred Reporting Items for Systematic Reviews and Meta-Analysis guidelines [18], and the protocol has been registered in the PROSPERO (No. CRD 42020158920). The electronic databases of PubMed, EmBase, and the Cochrane library were searched from their inception up to January 6^th^, 2022, and the core terms including (ticagrelor) and (polymorphism or allele or genotype or genetype or gene or SNP or genome or CYP2C19 or cytochrome P 450 CYP2C19 or cytochrome P-450 CYP2C19 or cytochrome P450 CYP2C19 or cytochrome P450CYP2C19 or CYPIIC19). The reference lists of retrieved studies were also reviewed manually to select any new eligible study. The detailed search strategy was in Additional file 1.

The literature search and study selection were independently undertaken by two authors, and the pilot test was used to refine and clarify eligibility criteria on ten to twelve papers. Conflicts between authors were settled by group discussion until a consensus was reached (κ = 0.81). The inclusion criteria were listed as follows: (1) all the included patients treated with ticagrelor; (2) the study should report PD and clinical outcomes of ticagrelor according to *CYP2C19* genotype (any loss-of-function (LOF) allele; or no LOF allele); (3) irrespective study reported qualitative or quantitative results; and (4) studies were only published in English. These with at least one LOF allele (*2 or *3) were classified as any LOF allele carriers, while those without any LOF allele were named no LOF allele carriers. MACEs were defined as composite of cardiovascular death, stroke, TIA, myocardial infarction (MI), and revascularization. Studies published as abstracts, animal experiments, PK and other language were excluded. The most comprehensive or most recent data were selected if the same cohort reported in multiple studies.

### Data collection and quality assessment

Two authors independently examined the included studies for the extracted data and quality assessment, and any disagreement was resolved by an additional author referring to original studies. The following information was collected into standardized tables: first author’s name, publication year, region, number of patients, mean age, male percentage, race, percentage of diabetes mellitus (DM), hypertension, dyslipidemia, smoking, MI, coronary artery bypass grafting (CABG), intervention, disease status, and reported outcomes. After this, the study quality was assessed by the Newcastle–Ottawa Scale (NOS), which was based on selection (4 items), comparability (1 item), and outcome (3 items) [19]. This scale assigned 0–9 points, and 7 or greater points were considered as high quality.

### Statistical analysis

The incidences of MACEs, MI, revascularization, stroke, definite stent thrombosis, bleeding, and high platelet reactivity were assigned as categories data, and the level of platelet reactivity was assigned as continuous data. The odds ratio (OR) and weighted mean difference (WMD) with corresponding 95% confidence interval (CI) were calculated through the random-effects model respectively [20, 21]. The heterogeneity across studies was evaluated with *Q* and *I*^2^ statistics, and we considered *I*^*2*^ > 50.0% or *P* values < 0.10 as indicative of significant heterogeneity [22, 23]. Subgroup analyses for exploring the heterogeneity were conducted based on ethnicity, sample size, smoking percentage, disease status and the NOS value. All statistical tests were two sided, and *P* value < 0.05 was regarded as statistical significance. Plot digitizer software was used to read the specify data in displayed figures, and the STATA software (Version 15.1; StataCorp, Texas, United States of America) was used for statistical analysis. In order to control the risk of type I and type II errors and calculate the required information size (RIS), trial sequential analysis (TSA) was performed using the TSA software (version 0.9.5.10 beta, http://www.ctu.dk/tsa) [24]. If the cumulative Z-curve stretched across the TSA monitoring boundaries or entered the RIS line, it was proved that a firm conclusion could be reached and no further studies were needed. The RIS was estimated using a = 0.05 (two sided) with 80% power.

## Results

### Literature search

**Fig. 1:**
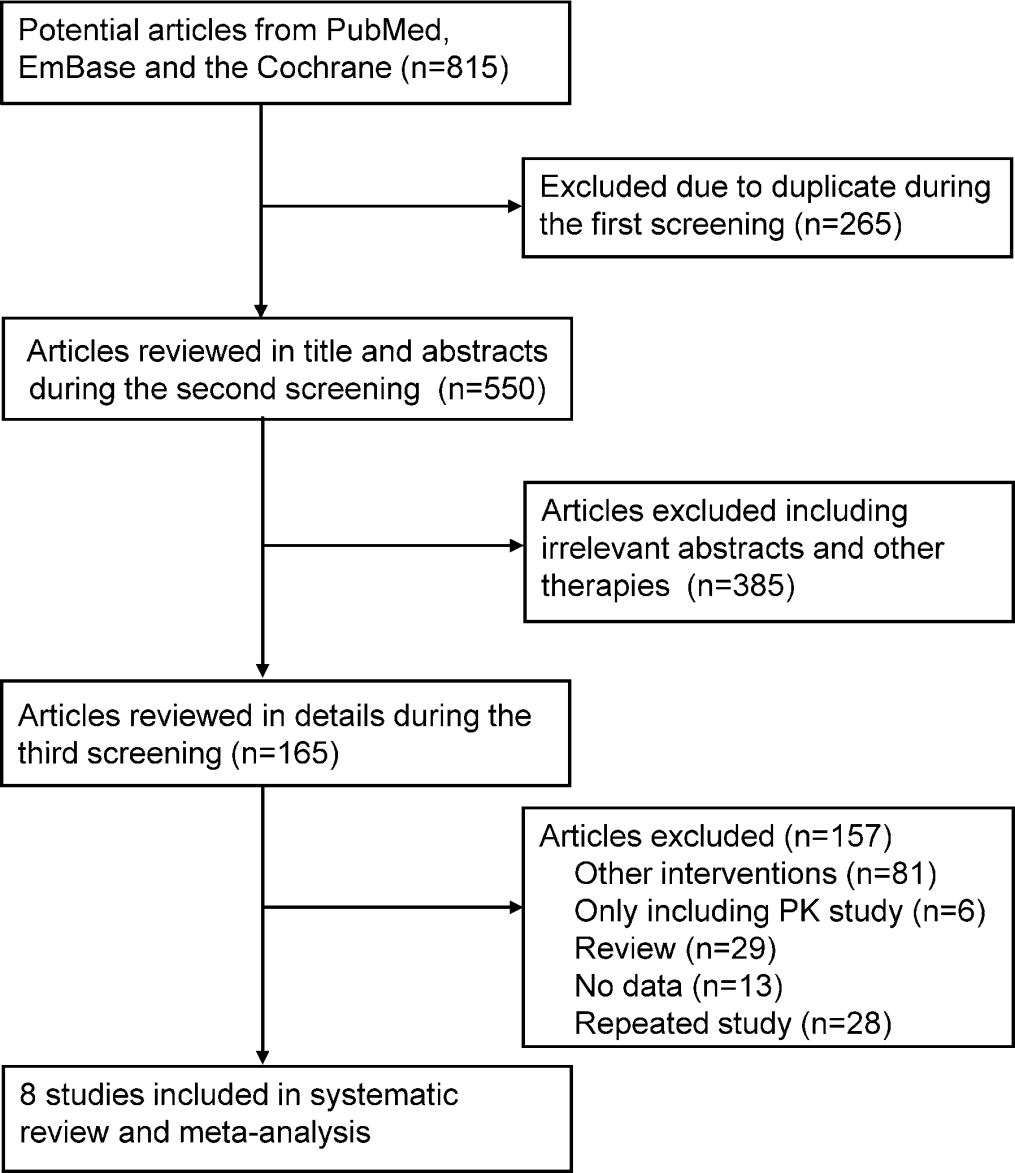
Flow diagram of the study selection process

The flow diagram of study selection process is shown in Fig. 1. 815 articles were identified in the initial electronic search (325 from PubMed, 321 from Embase, and 169 from the Cochrane Library); of which, 265 were excluded after removing duplicates. After excluding irrelevant abstracts or other therapies during the second screening, 385 studies were discarded. For the third screening, studies that reported other interventions (n = 81), only including pharmacokinetic study (n = 6), did not report available data (n = 13), or were review articles (n = 29) and repeated studies (n = 28) were further excluded. Eight studies were finally included into this systematic review [11–14, 25–28]. A manual search of the reference lists of these studies did not yield any new eligible studies.

### Study characteristics

Table 1 summarized the characteristics of included studies, which were all designed prospectively. The published years were ranged from 2010 to 2021, and 43 to 5137 patients were included in each study. The mean age ranged from 58.6 to 67.6 years, and male percentage ranged from 62.8 to 81.2%. The percentage of DM ranged from 23.0 to 38.8%, and percentage of smoking was 8.0 to 71.9%. Four studies were conducted in western countries, while the remaining were mainly conducted in China. One study quality was 8 points, 4 studies had 7 points and the remaining one had 5 points (Additional file 2).

**Table 1:** Characteristics of included studies

Author	Publication year	Region	No. of patients	Mean age (year)	Percentage of male (%)	Race (%)	DM (%)	Hypertension (%)	Dyslipidemia (%)	Smoking (%)	MI (%)	CABG (%)	Intervention	Disease status	Study quality
Tantry [25]	2010	USA	92	63.0	73.0	White (86.0)	24.0	84.0	92.0	8.0	47.0	40.0	Ticagrelor (180 mg load, 90 mg BID)	CAD	7
Wallentin [14]	2010	Multi-countries	5137	62.5	69.0	White (98.0)	23.0	NA	NA	35.0	NA	NA	Ticagrelor (90 mg BID)	ACS	7
Stimpfle [26]	2014	Germany	43	67.6	62.8	White (100.0)	38.8	NA	NA	38.5	81.4	2.6	Ticagrelor (180 mg load)	ACS	5
Dong [27]	2016	China	64	67.0	81.2	Asian (100.0)	32.8	48.4	48.4	71.9	NA	NA	Ticagrelor (180 mg load, 90 mg BID)	ACS	7
Wang [11]	2019	China	336	61.1	72.9	Asian (100.0)	23.5	60.4	6.0	47.6	NA	NA	Ticagrelor (180 mg load, 90 mg BID)	Acute minor stroke or TIA	7
Yu [12]	2020	China	247	NA	NA	Asian (100.0)	NA	NA	NA	NA	NA	NA	Ticagrelor (90 mg BID)	CAD	7
Machal [28]	2020	Czech	46	62.1	69.0	White (100.0)	24.0	61.0	28.0	59.0	NA	NA	Ticagrelor (180 mg load, 90 mg BID)	ACS	7
Zhang [13]	2021	China	440	58.6	78.9	Asian (100.0)	35.7	58.0	54.3	23.4	15.0	3.6	Ticagrelor (90 mg BID)	ACS after PCI	8

*ACS* acute coronary syndrome, *CAD* coronary artery disease, *DM* diabetes mellitus, *MI* myocardial infarction, *CABG* coronary artery bypass grafting, *NA* not available, *BID* twice daily, *TIA* transient ischemic attack, *PCI* percutaneous coronary intervention

### Qualitative analyses

The results of qualitative analyses were summarized in Table 2. Tantry et al. found *CYP2C19* genotype has no significant impact on antiplatelet effect of ticagrelor through three methods including aggregometry, VerifyNow P2Y12 and vasodilator-stimulated phosphoprotein-phosphorylation assay [25]. Wallentin et al. showed although the incidences of stroke, definite stent thrombosis, and bleeding in *CYP2C19* LOF allele carriers are higher than those with no LOF allele, whereas these increases were not statistically significant [14]. Stimpfle et al. found the platelet reactivity measured of adenosine diphosphate(ADP)-induced platelet aggregation in any LOF allele and no LOF allele of *CYP2C19* genotype were 12.27 ± 11.4 and 11.21 ± 7.0 AU*min (*P* > 0.05), respectively [26]. Dong et al. found no significant differences on the risk of death, MI, revascularization, and stroke according to *CYP2C19* genotype [27]. A study conducted by Wang et al. found patients with acute minor stroke or TIA carrying any *CYP2C19* LOF allele were associated with an increased risk of MACEs [11]. Yu et al. found bleeding complications were higher in patients carrying no *CYP2C19* LOF allele after PCI with coronary heart disease, while there was no difference in MACEs [12]. Machal et al. revealed that the ADP-induced platelet reactivity didn't differ among different *CYP2C19* genotype in ticagrelor-treated patients [28]. Finally, an ambispective single-center observational study conducted by Zhang et al. showed there was no significant difference in MACEs and bleeding between *CYP2C19* LOF group and non-LOF group of Chinese ACS patients after PCI [13].

**Table 2:** The investigated outcomes according to CYP2C19 genotype (Any LOF allele vs no LOF allele)

Author	MACE	MI	Revascularization	Stroke	Definite stent thrombosis	Bleeding	High platelet reactivity^3^	Platelet reactivity^4^
Tantry [25]	–	–	–	–	–	–	–	No statistical influence (data shown below)
Wallentin [14]	115/1384 (8.3%) vs 296/3554 (8.3%)	102/1384 (7.4%) vs 273/3554 (7.7%)^1^	–	13/1384 (0.9%) vs 23/3554 (0.6%)	15/943 (1.5%) vs 22/2341 (1.0%)	149/1380 (10.8%) vs 331/3547 (9.3%)	–	–
Stimpfle [26]	–	–	–	–	–	–	–	12.27 ± 11.4 vs 11.21 ± 7.0 AU*min
Dong [27]	13/38 (34.2%) vs 6/26 (23.1%)	3/38 (7.9%) vs 1/26 (3.8%)	3/38 (7.9%) vs 2/26 (7.7%)	4/38 (10.5%) vs 2/26 (7.7%)	–	–	–	–
Wang [11]	16/184(8.7%) vs 4/137 (2.9%)	–	–	15/184(8.2%) vs 4/137 (2.9%)	–	6/184 (3.3%) vs 6/137 (4.4%)^2^	17/157(10.8%) vs 16/118 (13.6%)	–
Yu [12]	23/202(11.4%) vs 7/45(15.6%)	0/202(0%) vs 0/45(0%)	–	0/202(0%) vs 0/45(0%)	0/202(0%) vs 0/45(0%)	27/202(13.4%) vs 16/45(35.6%)	–	–
Machal [28]	–	–	–	–	–	–	–	342 ± 267.2 vs 405 ± 385.2; 203 ± 64.5 vs 207 ± 96.3 AU*min
Zhang [13]	12/302(4.0%) vs 6/138(4.3%)	1/302(0.3%) vs 3/138(2.2%)	10/302(3.3%) vs 6/138(4.3%)	1/302(0.3%) vs 0/138(0%)	2/302(0.7%) vs 3/138(2.2%)	5/302(1.7%) vs 4/138(2.9%)	–	–

1: This result specifically included both cardiac death and myocardial infarction

2: This result specifically included both major and minor bleeding events

3: High platelet reactivity = P2Y12 reaction units of more than 208, as measured the VerifyNow P2Y12 assay

4: As methods of platelet reactivity assessment in these three studies were all different, we didn’t include these data for meta-analysis

Firstly, there were three methods of platelet reactivity assessment in Tantry’s study, including aggregometry, VerifyNow P2Y12 and vasodilator-stimulated phosphoprotein-phosphorylation (VASP) assay. All the methods were evaluated at 8 h postloading (A) and during maintenance phases (2 to 6 weeks, 8 h after the last dose) (B). The specify data and P value of LOF and no LOF groups at two timepoints were as follows respectively:

(i) 5 umol/L ADP-induced platelet aggregation (%): 19.94 ± 8.86 vs 18.10 ± 11.65, *P* = 0.518; 22.08 ± 10.93 vs 21.09 ± 12.00, *P* = 0.88

(ii) 20 umol/L ADP-induced platelet aggregation (%): 28.06 ± 9.85 vs 26.32 ± 12.61, *P* = 0.529; 29.01 ± 12.81 vs 29.01 ± 14.04, *P* = 0.803

(iii) P2Y12 Reaction Units: 41.19 ± 57.14 vs 43.04 ± 43.60, *P* = 0.301; 51.67 ± 52.56 vs 42.32 ± 36.97, *P* = 0.898

(iv) VASP-PRI (%): 24.16 ± 19.73 vs 20.10 ± 13.67, *P* = 0.616; 21.88 ± 15.11 vs 20.90 ± 16.02, *P* = 0.878

Secondly, the result of Stimpfle’s study was determined at earliest 2 h after loading (median 12 h) 180 mg of ticagrelor, while these results of Machal’s study were determined at 1 h after the first administration of ticagrelor and repeated after 24 h. Although both studies used the same method of Multiplate® analyzer (Roche), there was a big difference in values

*MACEs* major adverse cardiovascular events, *MI* myocardial infarction

### Quantitative analyses

**Fig. 2:**
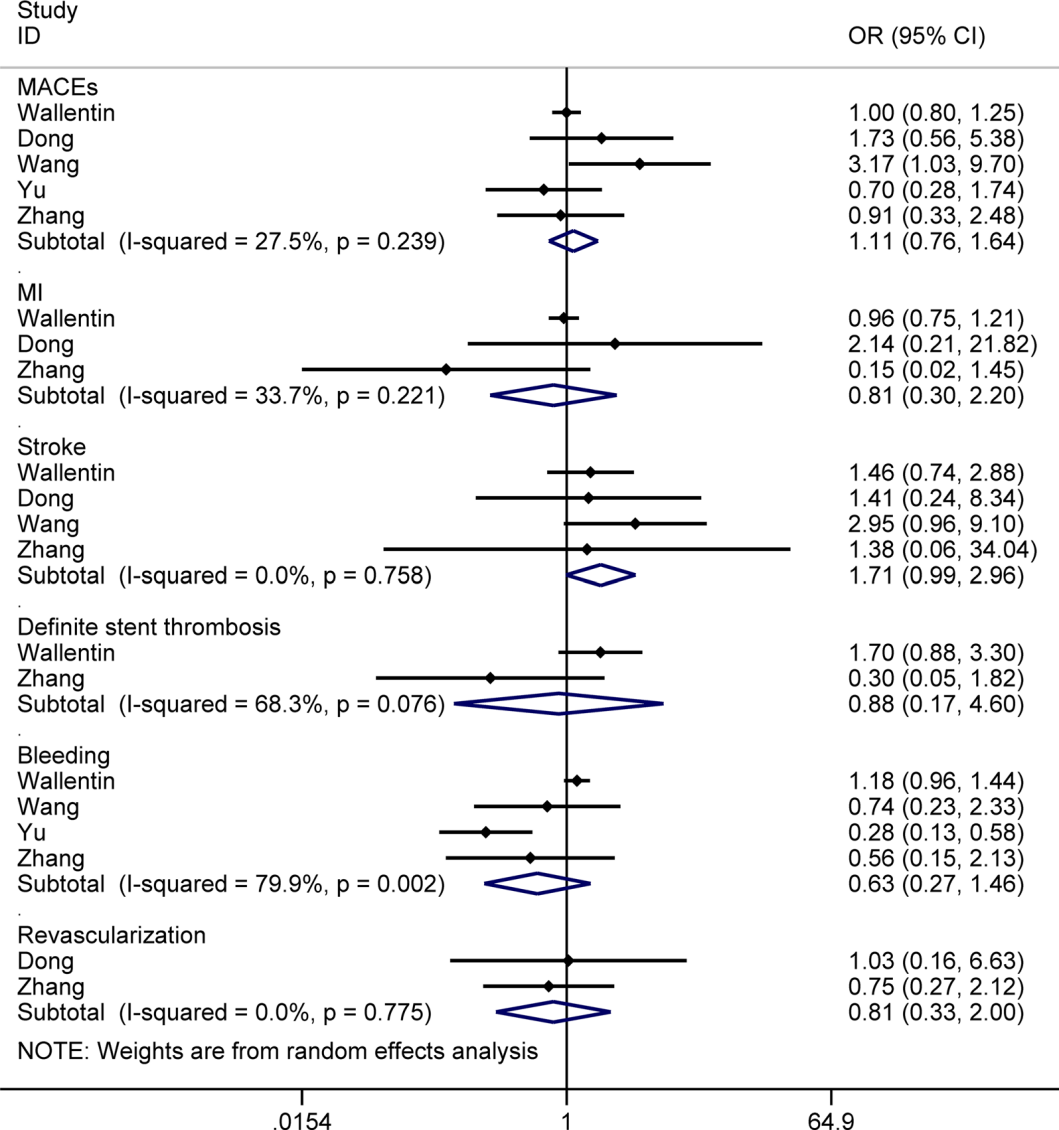
The quantitative results for the impacts of *CYP2C19* genotype on the pharmacodynamics and clinical outcomes of ticagrelor

The summary results for the impacts of *CYP2C19* genotype on PD and clinical outcomes of ticagrelor were shown in Fig. 2. Overall, although any *CYP2C19* LOF allele might affect the risk of MACEs (OR: 1.11; 95% CI: 0.76–1.64; *P* = 0.586) and stroke (OR: 1.71; 95% CI: 0.99–2.96; *P* = 0.054), whereas these associations without statistical significance. Furthermore, any *CYP2C19* LOF allele did not yield any impact on MI (OR: 0.81; 95% CI: 0.30–2.20; *P* = 0.682), definite stent thrombosis (OR: 0.88; 95% CI: 0.17–4.60; *P* = 0.882), bleeding (OR: 0.63; 95% CI: 0.27–1.46; *P* = 0.281), and revascularization (OR: 0.81; 95% CI: 0.33–2.00; *P* = 0.649). Significant heterogeneity was detected on the analysis of definite stent thrombosis and bleeding (*I*^2^ 68.3%, *P* = 0.076; *I*^2^ 79.9%, *P* = 0.002; respectively).

**Fig. 3:**
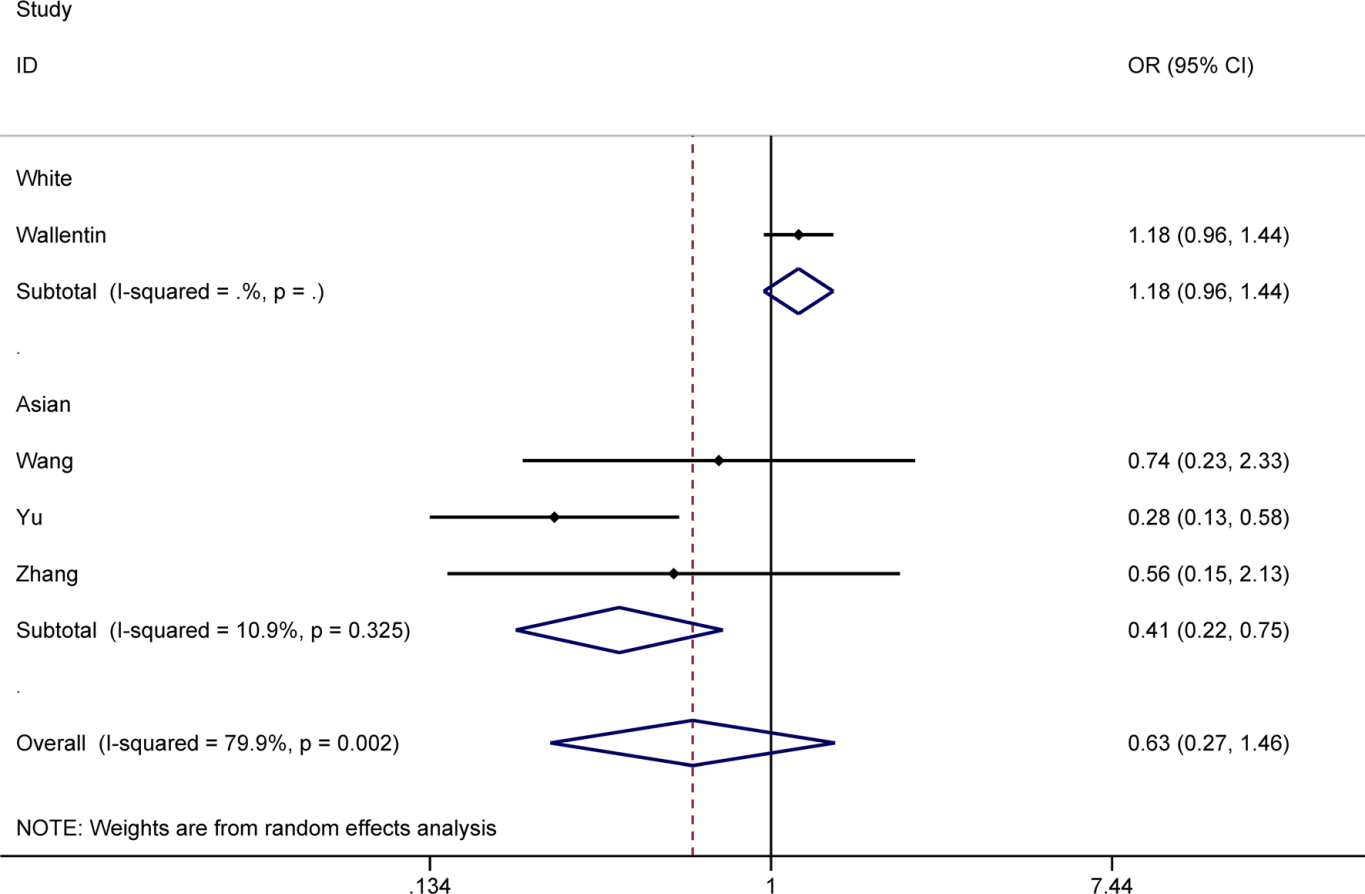
The subgroup analysis of ethnicity for the impacts of *CYP2C19* genotype on bleeding events of ticagrelor

To explore heterogeneity and more influencing factors, subgroup analyses were conducted according ethnicity, sample size, smoking percentage, disease status and NOS value. We noted Asian patients (small sample size) carrying any *CYP2C19* LOF allele were associated with a decreased risk of bleeding (OR: 0.41; 95% CI: 0.22–0.75; *P* = 0.004, Fig. 3), while the white race (large sample size) had no related association (OR: 1.18; 95% CI: 0.96–1.44; *P* = 0.120). Besides we found any LOF allele carriers diagnosed with stroke or TIA had an increased risk of MACEs (OR: 3.17; 95% CI: 1.03–9.07; *P* = 0.043), while patients diagnosed with ACS or coronary artery disease were not (OR: 0.99; 95% CI: 0.81–1.23; *P* = 0.953). However, the above subgroup of patients with stroke or TIA only included one study [11] and sample size was small, the result was worthy of further exploration. Finally, in any other subgroup analysis, we found no significant difference for *CYP2C19* genotypes in MI, revascularization, stroke, and definite stent thrombosis.

For MACEs, TSA showed the Z-curve did not cross either the conventional or trial sequential monitoring boundary, as well as the RIS (n = 27,900), which revealed that this effect remained no significance between two groups and more further trials were required (Additional file 3: Figure S1). For stoke, TSA found that the Z-curve crossed the conventional boundary, but not crossed the trial sequential monitoring boundary and RIS (n = 88,152), which suggested that the influence remained uncertain and more further trials were needed (Additional file 3: Figure S2). For the bleeding events of Asian subgroups, TSA also showed the similar results of stroke (RIS, n = 9585), which revealed that result of pooled subgroup analysis might be false positive and more further trials were required (Additional file 3: Figure S3). For the other outcomes, we performed TSA but the results didn’t show the trial sequential monitoring boundary and RIS, considering the sparse data and low event rates (Additional file 3: Figures S4–S7).

## Discussion

Our study mainly investigated the effect of *CYP2C19* polymorphism on PD (high platelet reactivity or platelet reactivity level) and clinical outcomes (MACEs, MI, revascularization, stroke, definite stent thrombosis, and bleeding) for patients treated with ticagrelor. To our knowledge, this is the first systematic review to focus this topic and provide both qualitative and quantitative results. Our review contained 8 studies and recruited a total of 6405 patients. The results might suggest any *CYP2C19* LOF allele of Asian patients might be associated with decreased risk of bleeding events, whereas the impacts on MACEs and stroke in all patients needed further large-scale prospective study to verify because of its non-significant increasing trend. Finally, the MI, revascularization, definite stent thrombosis and bleeding in all patients according to *CYP2C19* genotype were without statistical significance.

Although it was demonstrated that ticagrelor was superior to clopidogrel in reducing ischemic events in ACS patients [15] and the impact of *CYP2C19* genotype on the PD in clopidogrel have already illustrated in several studies [29, 30], while few study focused on *CYP2C19* polymorphism and the outcomes of ticagrelor. Most studies or meta-analysis mainly investigated on comparing newer P2Y12 inhibitors with clopidogrel for any *CYP2C19* LOF allele or no LOF allele carriers, and the beneficial effect was only observed in LOF allele carriers [31]. A subgroup analysis of 6 studies from a latest meta-analysis [32] showed that there was no significant difference of MACEs between patients with or without *CYP2C19* LOF alleles treated with newer P2Y12 inhibitors (RR 1.01; 95% CI 0.86–1.16; *P* = 0.94). Although this research didn’t distinguish prasugrel or ticagrelor, the above result was similar with our analysis focused on ticagrelor. Our pooled results might suggest the ischemic events of MACEs and stroke were not statistically significant, whereas patients carrying any *CYP2C19* LOF allele might present with an excess risk. These results needed further discussed as the smaller number of included studies and lower prevalence of outcomes. The TSA for MACEs and stroke also demonstrated the same results. Our accrued information size (n = 6010 for both) were far less than RIS (n = 27,900 for MACEs; n = 88,152 for stroke). Moreover, the above results could affect by several factors: the categories of MACEs in these studies were slightly different; the incidence of MACEs at various follow-up was varied; the therapies including invasive and non-invasive could biases the incidences [33]; the using of clopidogrel could affect the impact of *CYP2C19* genotype [34]. Therefore, these trends need further validation based on large scale prospective studies.

The meta-analysis conducted by Biswas et al. [32] also revealed there would be safe for using newer P2Y12 inhibitors among ACS patients undergoing PCI with *CYP2C19* LOF alleles. Interestingly, our subgroup analysis also found Asian patients carrying any *CYP2C19* LOF allele had a significant decreased risk of bleeding events compared with no LOF carriers. The studies conducted by Wang [11], Yu [12] and Zhang [13] et al. reported the opposite results in Chinese patients with ticagrelor. The benefit of reducing MACEs in any *CYP2C19* LOF allele carriers was lower than no LOF alleles carriers [11], while the benefit of reducing bleeding complications was higher [12]. A greater number of LOF alleles significantly increased the risk of ischemic events and decreased the risk of bleeding. This result might be meaningful for determining clinical antiplatelet therapy strategy for patients with *CYP2C19* genotype testing.

Recent evidences support the efficacy and safety of P2Y12 inhibitor monotherapy in preference of DAPT after coronary revascularization [35–37]. A meta-analysis [35] of six trials including 24,096 patients showed P2Y12 inhibitor monotherapy has significant lower risk of bleeding than DAPT, with a similar risk of death, MI, or stroke. For the primary study population, Asian population was the largest group (44.3%), and the ratio of P2Y12 inhibitor at randomization was ticagrelor 69.5%, prasugrel 1.0% and clopidogrel 29.5%, respectively. Interestingly, newer P2Y12 inhibitor monotherapy revealed the above benefit while clopidogrel monotherapy didn’t in subgroup analysis. Another recent meta-analysis [36] of eight trials including 37,775 patients was mainly focused on the impact of de-escalation of DAPT (D-DAPT, switching to P2Y12 inhibitor monotherapy, or dose reduction of P2Y12 inhibitor after 1 to 3 months) and 12 months standard DAPT (S-DAPT) after PCI among East Asians and non-East Asians. Compared with S-DAPT, the reduced risk of bleeding with D-DAPT was only demonstrated in East Asians but not in non-East Asians. Among different strategies of S-DAPT, the largest percent was ticagrelor monotherapy (75.2%), while the authors didn’t analyze the different effect of these strategies on clinical outcomes. These studies suggest that ticagrelor monotherapy after coronary revascularization has related advantages in reducing risk of bleeding, especially in Asian population. Combined with our findings, we can infer that ticagrelor is safer for *CYP2C19* LOF allele carriers. However, our TSA for bleeding events among Asian population revealed that results might be false positive and more further trials were required. The RIS of that TSA was equal to 9585, while our accrued information size was only 1008. As so far there are few studies reported the ethnic differences in the efficacy and safety of ticagrelor treatment. The study with single and multiple ascending doses of ticagrelor by Teng et al. [38] reported that the exposure in Japanese was greater in Caucasian healthy volunteers, while inhibition of platelet aggregation and bleeding time were similar. However, this study just included a small number of healthy volunteers and couldn’t completely reflect the ethnic differences. According to clinical pharmacogenetics implementation consortium data, the frequency of CYP2C19 *2 or *3 in Asian (29.0–34.3%, 0.9–8.3%) was higher than that in Caucasian (14.6%, 0.6%) [39]. Consequently, the risk of bleeding between *CYP2C19* genotypes in Asian patients should be interpreted with caution due to smaller number of people included, and more studies with large sample size needed to be verified.

Finally, the pooled results for MI, revascularization, and definite stent thrombosis according to *CYP2C19* genotype were available in smaller number of studies and were even not shown the trial sequential monitoring boundary and RIS by TSA performing, which needed further large-scale prospective study to verify. Besides, as evaluation PD indicators of ticagrelor, the platelet reactivity might be assessed by different methods [7, 40, 41]. Because each method reported its measurement index and the number of studies was indeed small, we couldn’t perform mate-analysis about PD and more future trials would be required.

The limitations of this systematic review should be acknowledged: (1) the patients’ characteristics could bias the prognosis of disease and affect the measured outcomes. These factors contained age, sex, body mass index, smoking, hypertension, high cholesterol, DM, planned invasive treatment, troponin positive, infarction site, diagnosis balloon dilatation time, and Killip grade of cardiac function [42–47]; (2) the whole number of studies included for quantitative analyses was only 5, and some outcomes only contained two studies. It was worthy of more exploring; (3) although the stratified analyses according to patients’ characteristics were conducted, the number of included studies was small and Asian patients included were far fewer than the white. And all Asian people came from China; (4) other antiplatelet therapies were not available from included studies, which could affect the progression of clinical outcomes [48]; (5) and the methodological evaluation of study quality was using NOS, and there was no comparability of cohorts with ticagrelor in all these studies, which might introduce uncontrolled biases and affect the reliable of results. Therefore, our findings should be recommended critically due to the quality of included studies and the difference of ethnicity.

## Conclusions

In summary, this study indicated *CYP2C19* genotype might play an important role on the risk of bleeding events in Asian patients treated with ticagrelor. There would be lower bleeding risk for Asians treated with ticagrelor carrying *CYP2C19* LOF alleles. Moreover, *CYP2C19* genotype had no significant impacts on MACEs, MI, stroke, revascularization, definite stent thrombosis, and bleeding in the whole cohort. Future large-scale prospective studies should be undertaken and more patients with different ethnicity should be included to verify these effects.

## Supplementary Information

**Additional file 1**. Search strategy of this systematic review and meta-analysis.

**Additional file 2**. The quality of the included studies assessed using the Newcastle–Ottawa Scale.

**Additional file 3**. Trial sequential analysis of pooled results of outcomes.

## Data Availability

All data generated or analysed during this study are included in this published article and its Additional files.

## Abbreviations

ACS: Acute coronary syndrome
CABG: Coronary artery bypass grafting
CI: Confidence interval
DAPT: Dual antiplatelet therapy
DM: Diabetes mellitus
LOF: Loss-of-function
MACEs: Major adverse cardiovascular events
MI: Myocardial infarction
NOS: Newcastle–Ottawa Scale
OR: Odds ratio
PCI: Percutaneous coronary intervention
PROSPERO: International Prospective Register of Ongoing Systematic Reviews
RIS: Required information size
SOCRATES: Acute Stroke or Transient Ischaemic Attack Treated With Aspirin or Ticagrelor and Patient Outcomes
TIA: Transient ischemic attack
TSA: Trial sequential analysis
WMD: Weight mean difference
