# Supplementary material for: Effect of CYP2C19 genetic polymorphism on the pharmacodynamics and clinical outcomes for patients treated with ticagrelor: a systematic review with qualitative and quantitative meta-analysis
Source: BMC Cardiovasc Disord. 2022 Mar 17;22:111. doi: 10.1186/s12872-022-02547-3 (PMC8928616; doi:10.1186/s12872-022-02547-3)
Supplement: Supplementary file 1 — Additional file 1. Search strategy of this systematic review and meta-analysis. [file 12872_2022_2547_MOESM1_ESM.docx]

**Supplemental 1**

Effect of *CYP2C19* genetic polymorphism on the pharmacodynamics and clinical outcomes for patients treated with ticagrelor: a systematic review with qualitative and quantitative meta-analysis

Search terms in each data base as follows:

1. Pubmed：
(ticagrelor) and (polymorphism or allele or genotype or genetype or gene or SNP or genome or CYP2C19 or cytochrome P 450 CYP2C19 or cytochrome P-450 CYP2C19 or cytochrome P450 CYP2C19 or cytochrome P450CYP2C19 or CYPIIC19)

2. Cochrane（CENTRAL）：
("ticagrelor":ti,ab,kw) and ("polymorphism":ti,ab,kw or "allele":ti,ab,kw or "genotype":ti,ab,kw or "genetype":ti,ab,kw or "gene":ti,ab,kw or "SNP":ti,ab,kw or "genome":ti,ab,kw or "CYP2C19":ti,ab,kw or "cytochrome P 450 CYP2C19":ti,ab,kw or "cytochrome P-450 CYP2C19":ti,ab,kw or "cytochrome P450 CYP2C19":ti,ab,kw or "cytochrome P450CYP2C19":ti,ab,kw or "CYPIIC19":ti,ab,kw)

3. EMBASE：
(ticagrelor:ab,ti) AND (polymorphism:ab,ti OR allele:ab,ti OR genotype:ab,ti OR genetype:ab,ti OR gene:ab,ti OR SNP:ab,ti OR genome:ab,ti OR CYP2C19:ab,ti OR "cytochrome P 450 CYP2C19":ab,ti OR "cytochrome P-450 CYP2C19":ab,ti OR "cytochrome P450 CYP2C19":ab,ti OR "cytochrome P450CYP2C19":ab,ti OR CYPIIC19:ab,ti )
