# Supplementary material for: Effect of CYP2C19 genetic polymorphism on the pharmacodynamics and clinical outcomes for patients treated with ticagrelor: a systematic review with qualitative and quantitative meta-analysis
Source: BMC Cardiovasc Disord. 2022 Mar 17;22:111. doi: 10.1186/s12872-022-02547-3 (PMC8928616; doi:10.1186/s12872-022-02547-3)
Supplement: Supplementary file 2 — Additional file 2. The quality of the included studies assessed using the Newcastle–Ottawa Scale. [file 12872_2022_2547_MOESM2_ESM.docx]

**Supplemental 2**

Table. The quality of the included studies assessed using the Newcastle–Ottawa Scale

|  | **Selection** | | | |  | **Comparability** |  | **Outcome** | | |  |
| --- | --- | --- | --- | --- | --- | --- | --- | --- | --- | --- | --- |
| Study | Representativeness of the exposed cohort | Selection of the non exposed cohort | Ascertainment of exposure | Demonstration that outcome of interest was not present at start of study |  | Comparability of cohorts on the basis of the design or analysis |  | Assessment of outcome | Was follow-up long enough for outcomes to occur | Adequacy of follow up of cohorts | Scores |
| Tantry [25] | 1 | 1 | 1 | 1 |  | 0 |  | 1 | 1 | 1 | 7 |
| Wallentin [14] | 1 | 1 | 1 | 1 |  | 0 |  | 1 | 1 | 1 | 7 |
| Stimpfle [26] | 1 | 1 | 1 | 1 |  | 0 |  | 1 | 0 | 0 | 5 |
| Dong [27] | 1 | 1 | 1 | 1 |  | 0 |  | 1 | 1 | 1 | 7 |
| Wang [11] | 1 | 1 | 1 | 1 |  | 0 |  | 1 | 1 | 1 | 7 |
| Yu[12] | 1 | 1 | 1 | 1 |  | 0 |  | 1 | 1 | 1 | 7 |
| Machal[28] | 1 | 1 | 1 | 1 |  | 0 |  | 1 | 1 | 1 | 7 |
| Zhang [13] | 1 | 1 | 1 | 1 |  | 1 |  | 1 | 1 | 1 | 8 |
