# Supplementary material for: Effect of CYP2C19 genetic polymorphism on the pharmacodynamics and clinical outcomes for patients treated with ticagrelor: a systematic review with qualitative and quantitative meta-analysis
Source: BMC Cardiovasc Disord. 2022 Mar 17;22:111. doi: 10.1186/s12872-022-02547-3 (PMC8928616; doi:10.1186/s12872-022-02547-3)
Supplement: Supplementary file 3 — Additional file 3. Trial sequential analysis of pooled results of outcomes. [file 12872_2022_2547_MOESM3_ESM.docx]

**Supplemental 3**

**Figure S1**. Trial sequential analysis of MACEs


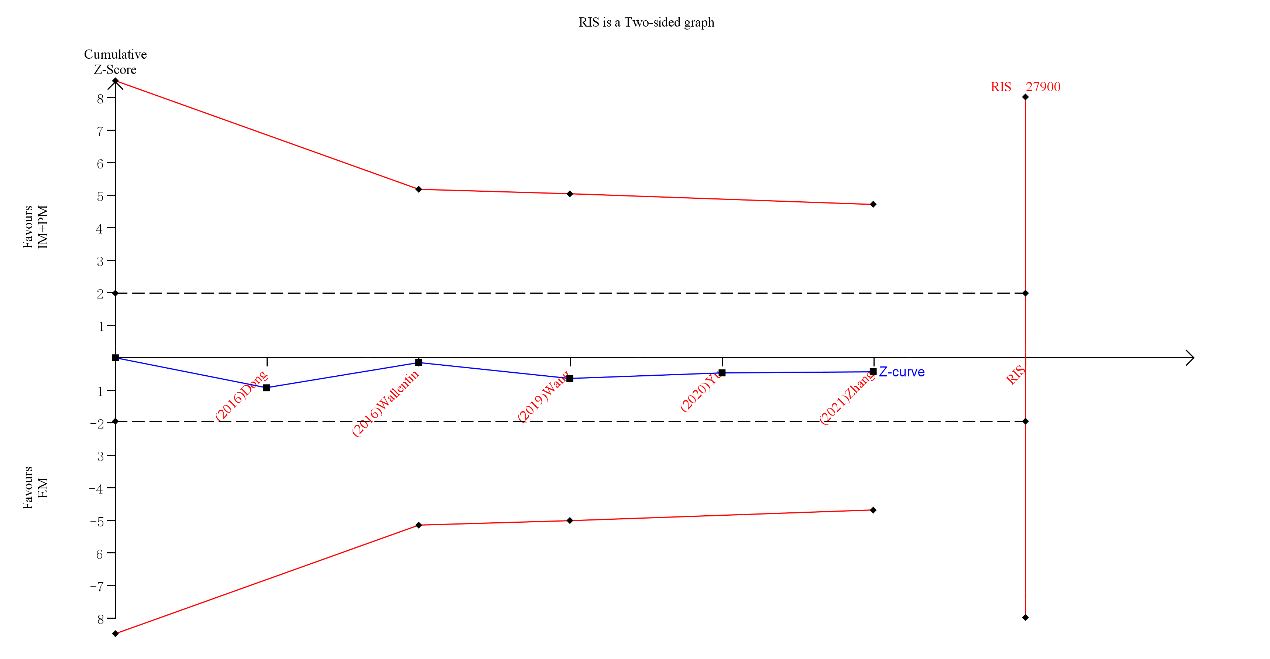


**Figure S2**. Trial sequential analysis of stoke


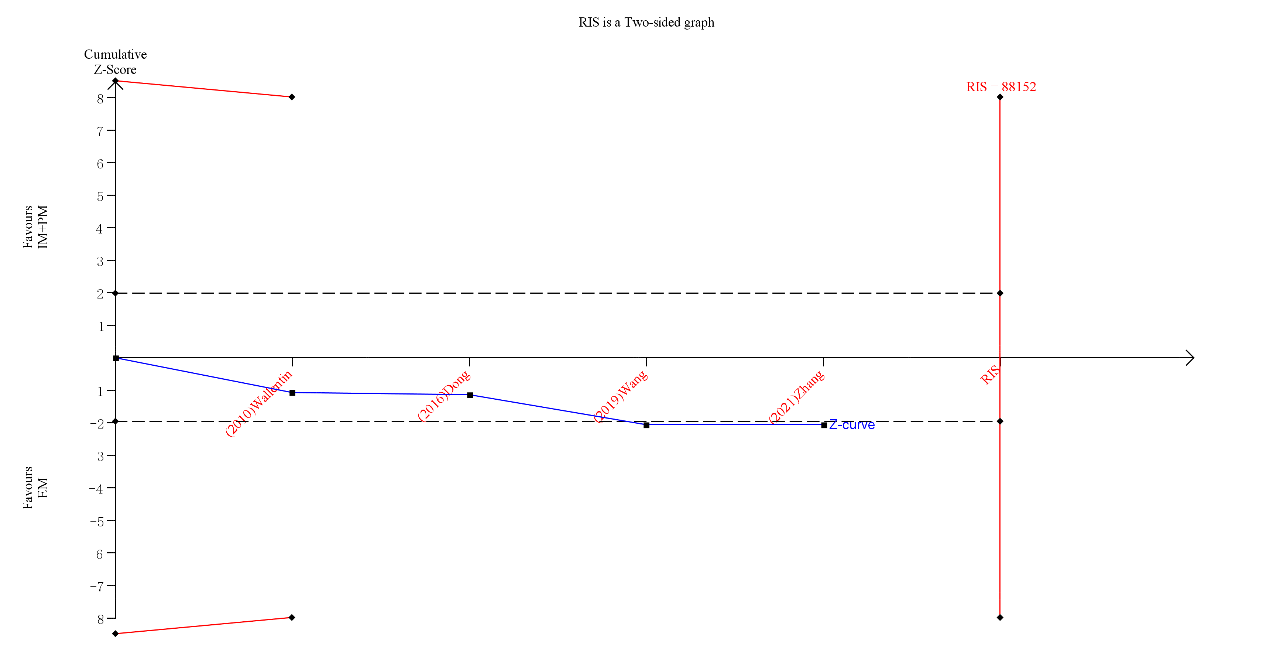


**Figure S3**. Trial sequential analysis of the bleeding events of Asian subgroup


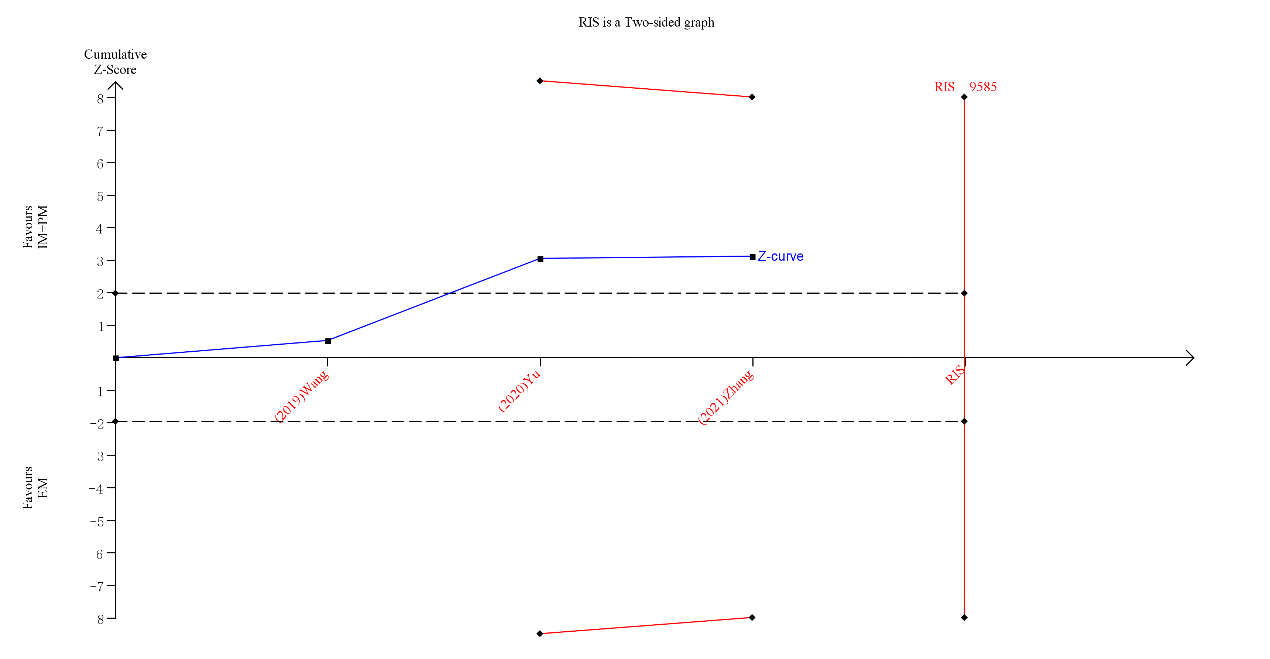


**Figure S4**. Trial sequential analysis of the bleeding events


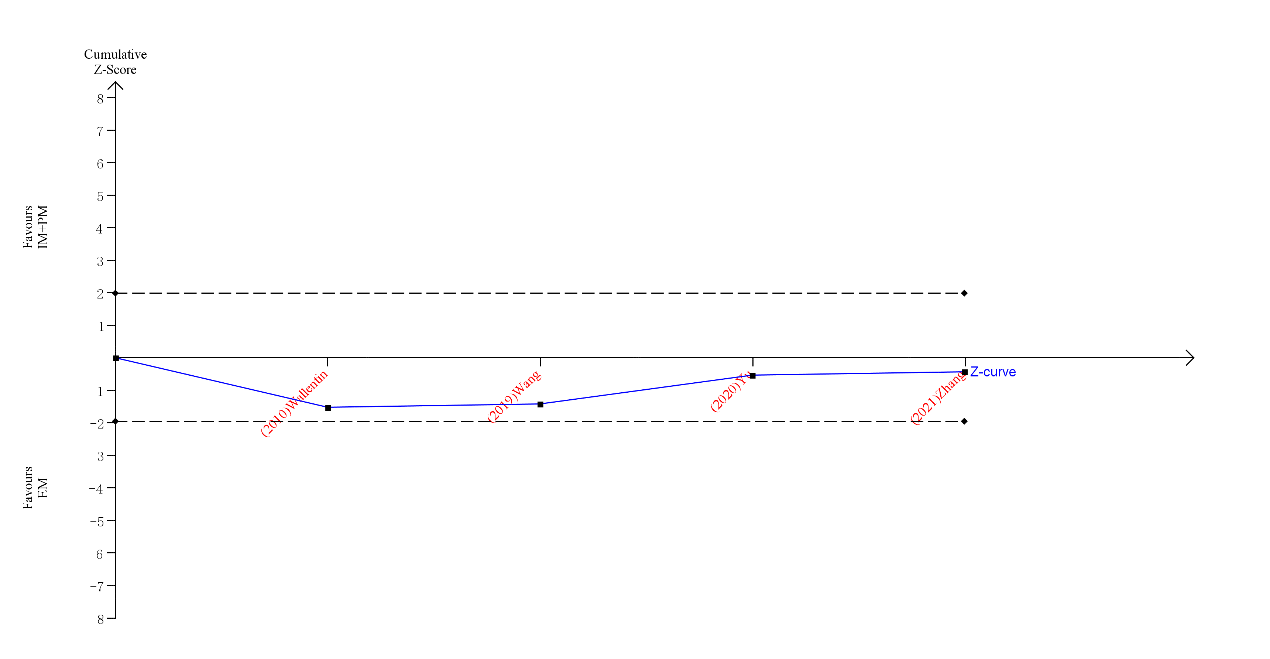


**Figure S5**. Trial sequential analysis of MI


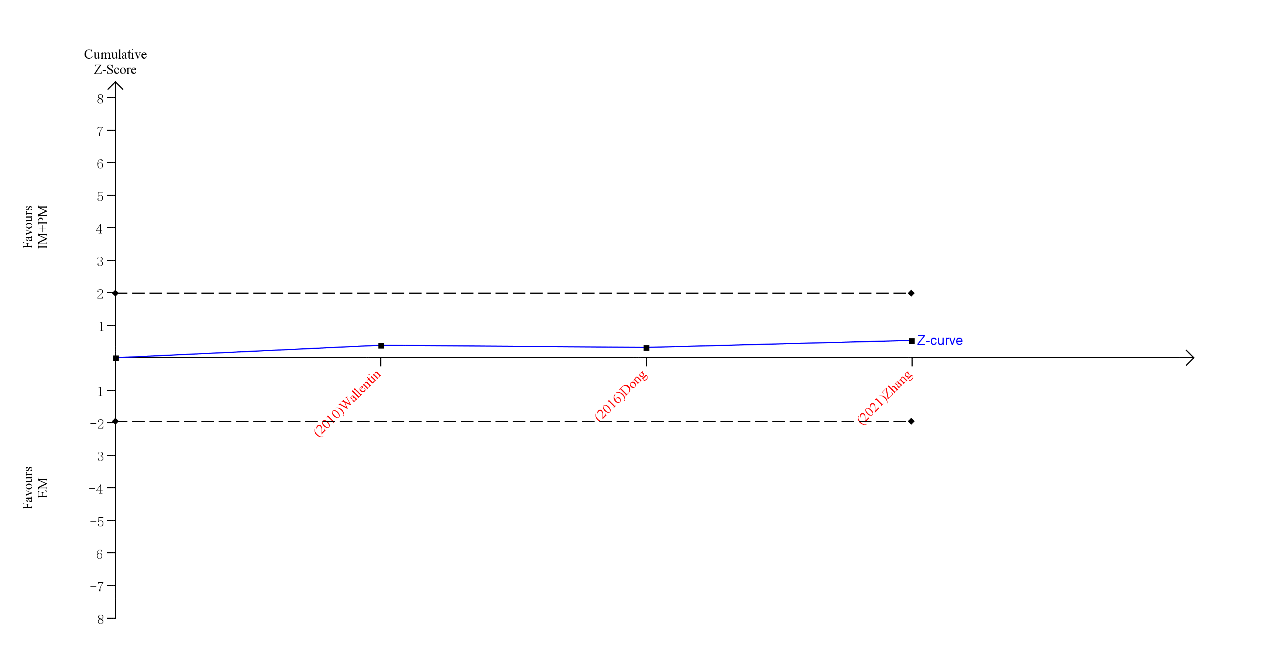


**Figure S6**. Trial sequential analysis of definite stent thrombosis


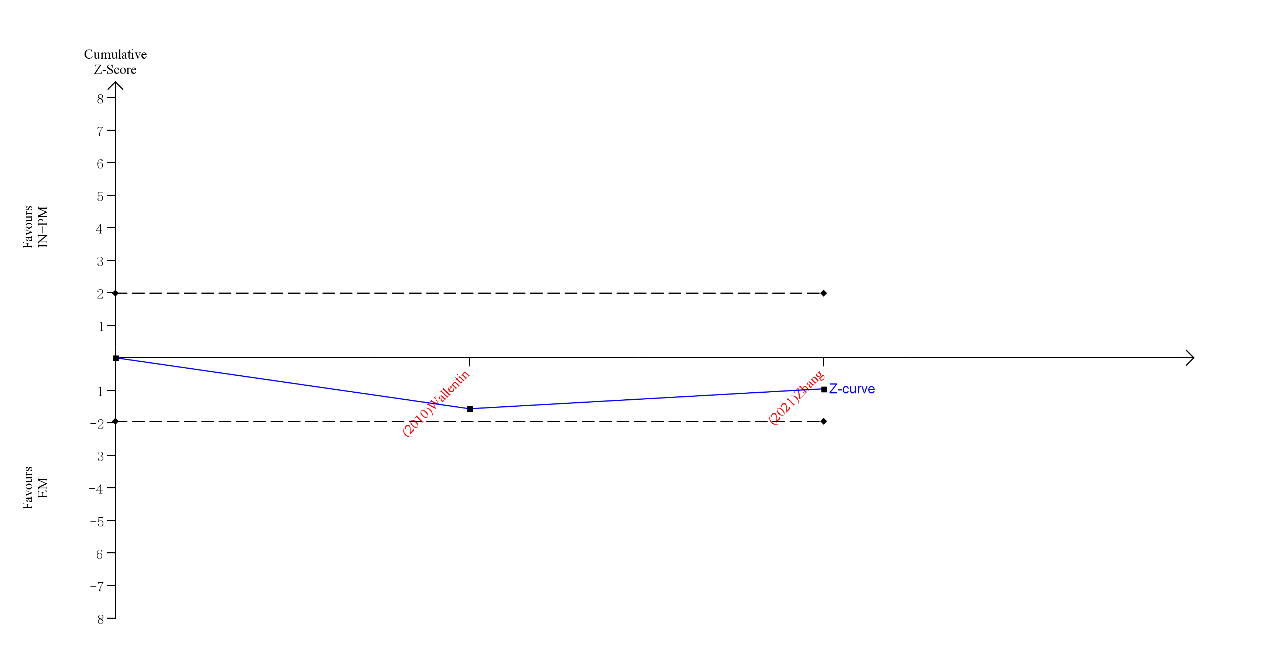


**Figure S7**. Trial sequential analysis of revascularization


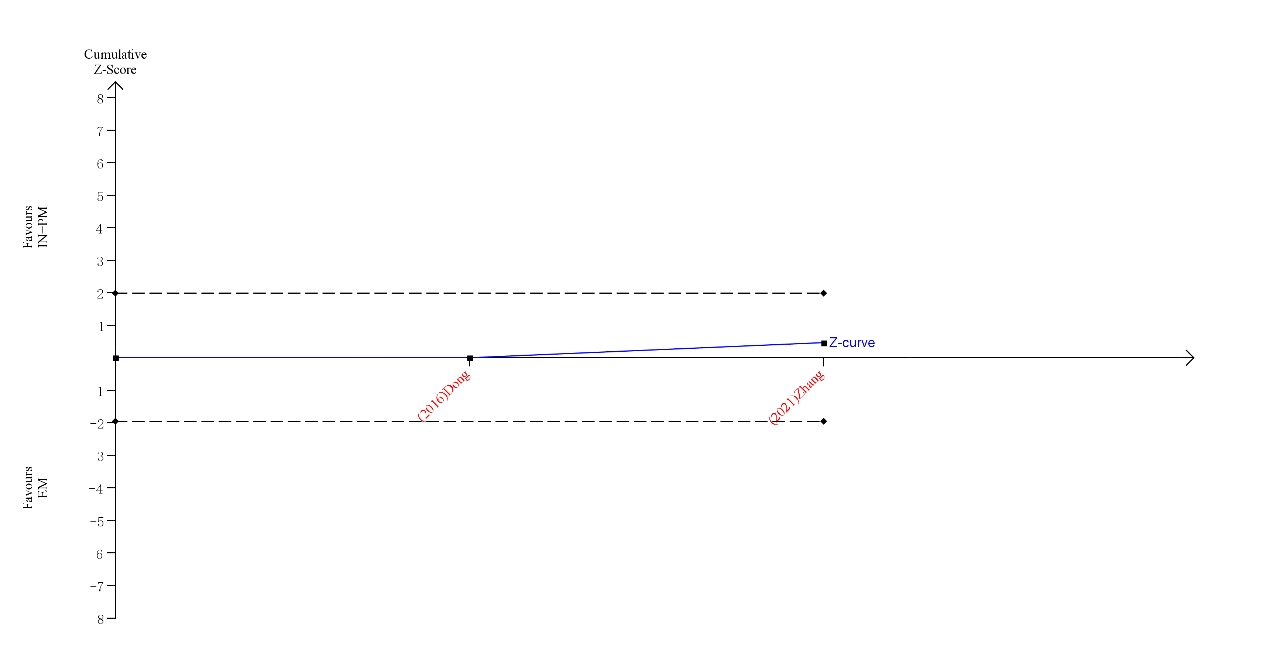


Annotation of all these figures:

TSA for a relative risk improvement of 20%. The continuous blue line represents the Z line (cumulative effect size), red lines represent the trial sequential monitoring boundaries, and required information size (RIS, the estimated optimal sample size adjusted to sample size and repeated analysis). The black dashed lines represent the conventional CIs.
